# Doppler evaluation of hepatic hemodynamics after living donor liver transplantation in infants

**DOI:** 10.3389/fbioe.2022.903385

**Published:** 2022-08-11

**Authors:** Xiping Chen, Huan Xiao, Chunjiang Yang, Jingyu Chen, Yang Gao, Yi Tang, Xiaojuan Ji

**Affiliations:** ^1^ Department of Ultrasound, National Clinical Research Center for Child Health and Disorders, Ministry of Education Key Laboratory of Child Development and Disorders, Children’s Hospital of Chongqing Medical University, Chongqing, China; ^2^ Department of Ultrasound, Chongqing General Hospital, Chongqing, China

**Keywords:** Doppler ultrasound, hemodynamics, liver transplantation, hepatic artery, portal vein, child

## Abstract

**Objective:** The aim of this study was to explore the hemodynamic changes of hepatic artery and portal vein detected by Doppler ultrasound (DU) in infants who underwent living donor liver transplantation (LDLT).

**Methods:** The data of 41 infant patients (22 Males, 19 Females, median age of 5 months) were collected in the Children’s Hospital affiliated to the Chongqing Medical University from May 2018 to December 2019. The patients underwent left lateral segment LDLT (LLS -LDLT) because of biliary atresia (BA). Hemodynamic parameters, including the peak systolic velocity (PSV), resistivity index (RI) of the hepatic artery (HA), portal vein velocity (PVV), and portal vein flow (PVF) were recorded from Doppler ultrasound on the day before the operation, and on the 1st, the 7th, the 14th and the 30th day after LDLT procedures. The changes of PSV_HA_, RI_HA_, PVV and PVF before and on the 1st day after transplantation were analyzed by paired *t*-test. The comparison of the data between different postoperative time points were assessed by ANOVA.

**Results:** Compared with the parameters measured before LDLT, PSV_HA_, and RI_HA_ decreased, and PVV and PVF increased significantly (*p* < 0.001) on the 1st day after LLS-LDLT. As for PSV, there was no significant difference between the 7th day and the 1st day after transplantation (POD7 *VS* POD1, *p* = 0.167) while there was a substantial difference between the 14th, 30th and 1st day after LT (POD14 vs*.* POD1, *p* = 0.003) (POD30 vs*.* POD1, *p* ＜0.001). And there was a significant difference between the 14th, 30th, and 7th days after LT (POD14 vs*.* POD7, *p* = 0.014) (POD30 vs*.* POD7, *p* ＜0.001). There was no significant difference between 30th and 14th after transplantation (POD30 vs*.* POD14, *p* = 0.092). As for RI_HA_ and PVV, the decrease was slow within the first month after the operation, and there was no significant difference at different times.

**Conclusion:** We have identified major hepatic flow changes that occurred in 41 infants who underwent LLS -LDLT due to BA. The data could be used for future studies of LDLT in infants including hemodynamic modeling, liver regeneration and clinical management.

## Introduction

Biliary atresia (BA) is a congenital liver disease with abnormal development of intra-hepatic and extra-hepatic bile ducts as its main pathological features. If untreated, BA often rapidly progresses to cholestatic cirrhosis and death before the age of 2 years (Hartley et al., 2009). BA remains the most common indication for liver transplantation in children (Wang et al., 2013; Wan et al., 2016), accounting for about 50% of transplantation cases in the United States, 74% in Europe, and 80% in Taiwan (Kelly et al., 2013). In recent years, Live donor liver transplantation (LDLT) has been the primary way of pediatric liver transplantation. LDLT has been successfully carried out in countries such as China and Japan where cadaveric donations are scant for cultural or religious reasons, and is increasingly used in other countries (Chen et al., 2006).

Understanding the regular changes in postoperative hemodynamics is helpful to detect vascular complications, and it can improve the survival rate after liver transplantation (Piardi et al., 2016). As a complex parenchymal organ, the liver is uniquely perfused by a dual-supply system, i.e., from the portal vein and hepatic artery: approximately 75% of the blood supply comes from the portal vein and 25% from the hepatic artery. In addition, there is an intimate relationship between the two vascular systems, termed the hepatic arterial buffer response (HABR) for the first time in 1981 by Lautt. With HABR, the hepatic arterial flow increases when the portal blood flow reduces, and the hepatic arterial flow decreases when the portal flow increases to maintain a stable blood supply to the liver (Lautt et al., 1990; Jakab et al., 1995; Sanada et al., 2011).

Doppler ultrasound (DU) can evaluate the graft blood supply and the patency of graft vessels in real-time. It has played a vital role in providing a reliable imaging basis for the early identification of vascular complications, making it a preferred imaging modality for monitoring the changes in hemodynamics after liver transplantation. Previous studies used Doppler ultrasound to detect vascular complications after liver transplantation (Someda et al., 1995; Abdelaziz and Attia, 2016; Low et al., 2013). For example, a study by Jamieson et al. (2014) revealed the hepatic hemodynamic changes less than 48 h after liver transplantation in a group of children under 14 years of age (the median age at transplantation was 1.3 years, with the youngest 25 days old and oldest 14 years old). By investigating the Doppler parameters at postoperative day (POD) 1, the study showed that a higher average velocity and resistive index, and low PSV_HA_, RI_HA_, and PVV are associated with vascular complications. Tang et al. (2021) analyzed the changing trend of hemodynamic parameters in uncomplicated children after liver transplantation. They concluded that the transplanted liver was initially hyperdynamic with high velocities at POD1. Their study also showed that the normal reference ranges of the artery and portal venous flow velocity varied widely during the 1st week after liver transplantation.

Since the most common vascular complications after liver transplantation are related to hepatic artery and portal vein, and acute vascular complications mainly occur within 1 month post-operation, this article aims to investigate these hemodynamic changes of the hepatic artery and portal vein in a cohort of infants receiving LDLT due to BA.

## Materials and methods

### Study population

The ethics committee of the Children’s hospital affiliated to the Chongqing Medical University approved this study. The data of the 41 pediatric patients (22 males, 19 females) who received LLS -LDLT because of biliary atresia (BA) were collected. The surgeries were performed between May 2018 and December 2019 by the same surgeon with more than 12 years of LDLT surgical experience. The patients aged between 4 and 18 months at the time of liver transplantation, with a median age of 5 months. The livers of the BA patients had biliary cirrhosis (stageⅣ) according to postoperative pathological analysis. The postoperative hospitalization days ranged from 17 to 65 days (mean ± SD 29.46 ± 10, median 29 days). The patients underwent LLS -LDLT with their parents as the live liver donors. Exclusion criteria included vascular complications and biliary complications within 3 months after transplantation, transplant rejection, and incomplete clinical data. Only these patients who met the above criteria were enrolled in the study. All children had been followed up for a period of median 8 months (range 3–16 months) after transplantation.

At the beginning of the study, there were 56 patients who underwent LLS-LDLT. From this cohort, 15 cases were excluded: five children with other original liver diseases instead of BA; four children had vascular complications (three cases of portal vein thrombosis on POD1, one case of portal vein stenosis on POD33), two children had anastomotic biliary stricture (ABS) in the first month after transplantation, two children had acute rejection and two children with incomplete clinical data.

### Doppler ultrasound examination

For preoperative Doppler ultrasonography, the HITACHI ALOKA ARIETTA 70 ultrasound scanner with a 2–5 MHz convex probe and the portable Mindray M9 ultrasound scanner with a 3–5 MHz convex probe were used for detecting the preoperative hepatic hemodynamic parameters. Preoperative ultrasound is usually performed 1 day before liver transplantation. The portable Mindray M9 ultrasound scanner with a 3–5 MHz convex probe was used for postoperative examination. Postoperative ultrasound examinations were recorded on the 1st, 7th, 14th, and 30th days after liver transplantation (POD1, POD7, POD14, and POD30). Patients were examined at the supine position in a quiet state. A multi-section scan is performed under the costal margin, intercostal space, or xiphoid process during an ultrasound examination. In all velocity measurements, the angle between the Doppler beam and blood flow direction was kept smaller than 60°.

The oblique section of the left portal vein of the donor site of the graft was chosen for ultrasound parameter measurement. We detect the blood flow velocity of the portal vein at the widest part of the main portal vein at the hilar hepatis preoperation, and at a distance of 1–2 cm from the portal vein anastomosis postoperation. We also measured the velocity of the hepatic artery nearby the portal vein at the porta hepatis. The portal vein diameter (D) where the velocity was measured was collected on the day before the operation and on the 1st day after LDLT. The parameters including the peak systolic velocity (PSV), resistive index (RI) of the hepatic artery, the portal vein diameter (D) and portal vein velocity (PVV) were collected. The RI_HA_ was calculated according to the formula: (peak systolic velocity- peak end-diastolic velocity)/peak systolic velocity. The portal vein flow (PVF) was calculated according to the formula: PVF = π × r^2^ × 0.57PVV × 60 [r: D/2 (cm), PVV: portal vein velocity (cm/s)], the unit of PVF is ml/min/100 g. All examinations were initially performed by two ultrasonologists with 5–8 years of DU imaging experience. They reviewed ultrasound images in consensus.

### Statistical analysis

Using the SPSS 19.0 statistical software, the blood flow parameters of the hepatic artery and portal vein before liver transplantation and the first day after the operation were compared using the paired *t*-test. The differences in the Doppler parameters between each time point after transplantation were analyzed by repeated-measures analysis of the variance. *p* < 0.05 indicated the difference was statistically significant.

## Results

### Comparison of hemodynamic parameters before and 1 day after liver transplantation

**FIGURE 1:**
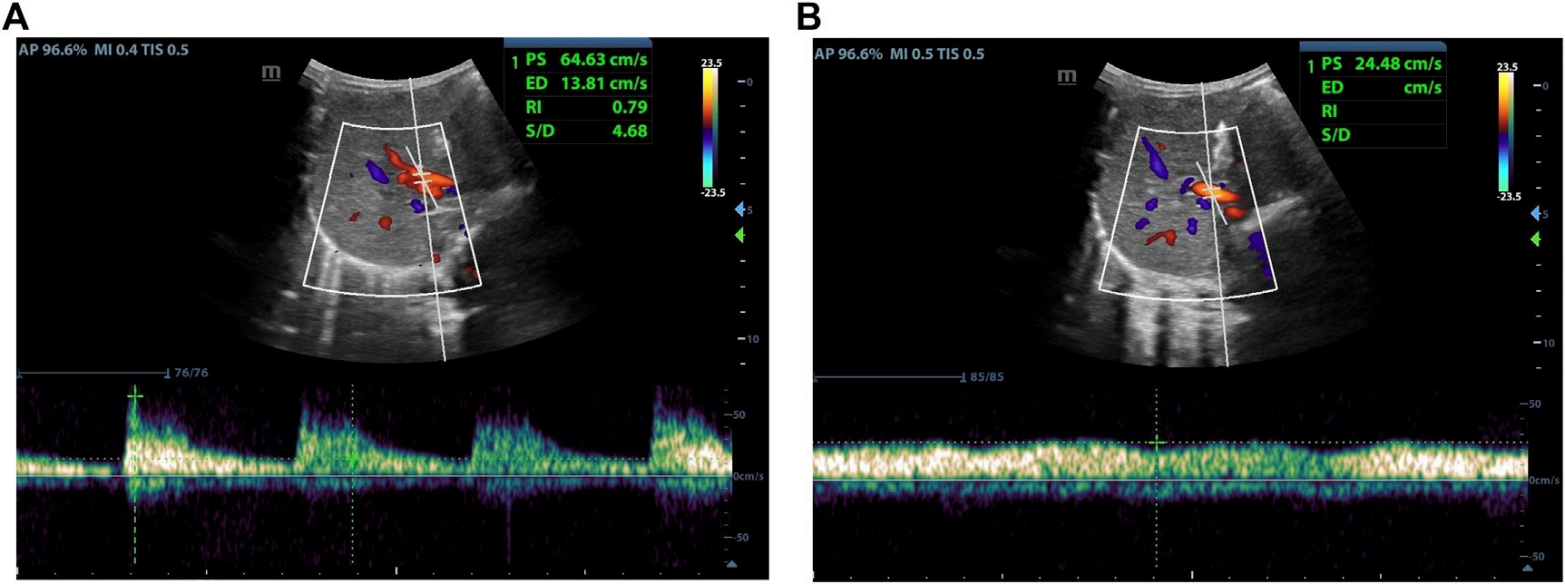
Female, 5 months old,1 day before LDLT. **(A)** Hepatic artery flow spectrum. **(B)** Portal vein flow spectrum.

**FIGURE 2:**
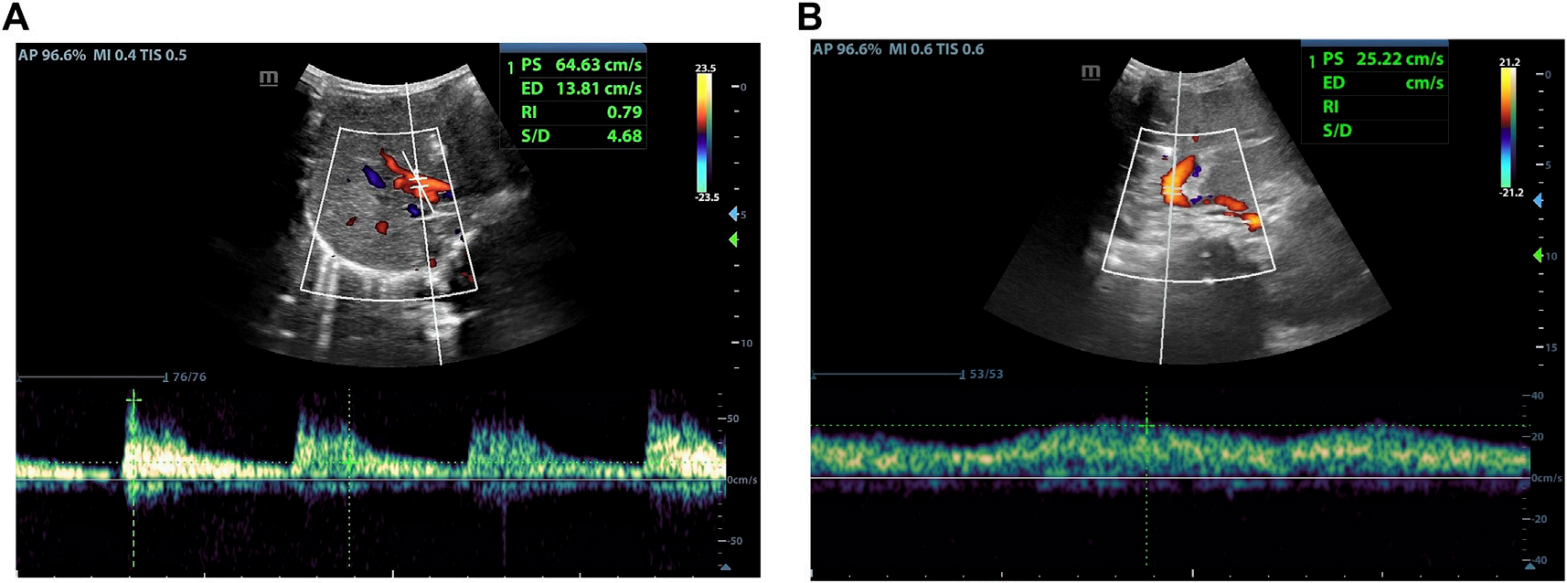
Female, 18 months old, 1 day after LDLT. **(A)** Hepatic artery flow spectrum. **(B)** Portal vein flow spectrum.

As indicated in Table 1, analyze the parameters of hepatic artery and portal vein before and 1 day after LDLT, including PSV, RI, PVV, and PVF, in children who have undergone LLS -LDLT. It was found that the value of hepatic artery PSV and RI dropped significantly on POD1, compared with that of the day before liver transplantation, while PVV and PVF went higher (Figures 1, 2).

**TABLE 1:** Parameters of hepatic artery and portal vein before and 1 day after LDLT (*x* ± s).

Time	PSV (cm/s)	RI	D (cm)	PVV (cm/s)	PVF (ml/min/100 g)
Before LDLT	73.32 ± 22.31	0.77 ± 0.09	0.44 ± 0.09	16.88 ± 5.69	83.87 ± 42.81
After LDLT	53.10 ± 16.02^a^	0.61 ± 0.05^a^	0.46 ± 0.09	30.80 ± 8.67^a^	165.99 ± 68.15^a^

Compared with before LDLT, ^a^ *p* < 0.01.

PSV, peak systolic velocity of the hepatic artery; RI, resistive index of the hepatic artery.

PVV, portal vein velocity; PVF, portal vein flow; D, portal vein diameter.

### Comparison of blood flow parameters between each time point within 1 month after transplantation

**FIGURE 3:**
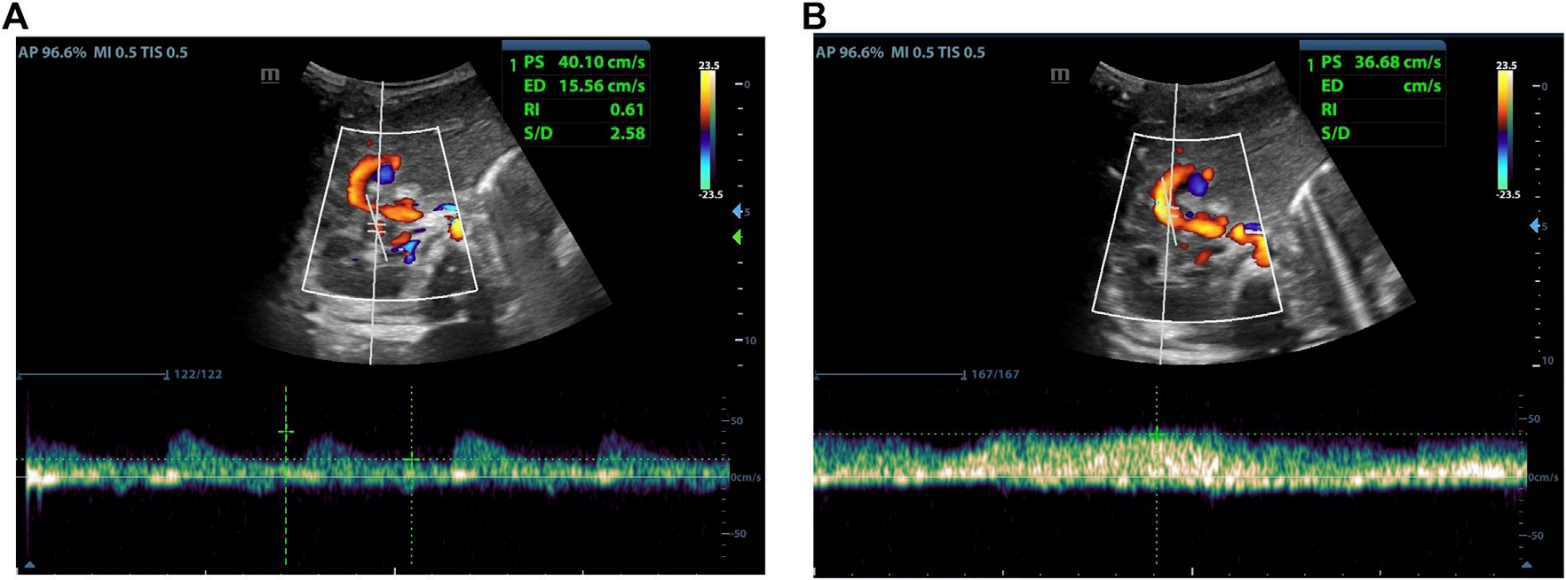
Female, 4 months old, 7 days after LDLT. **(A)** Hepatic artery flow spectrum. **(B)** Portal vein flow spectrum.

**FIGURE 4:**
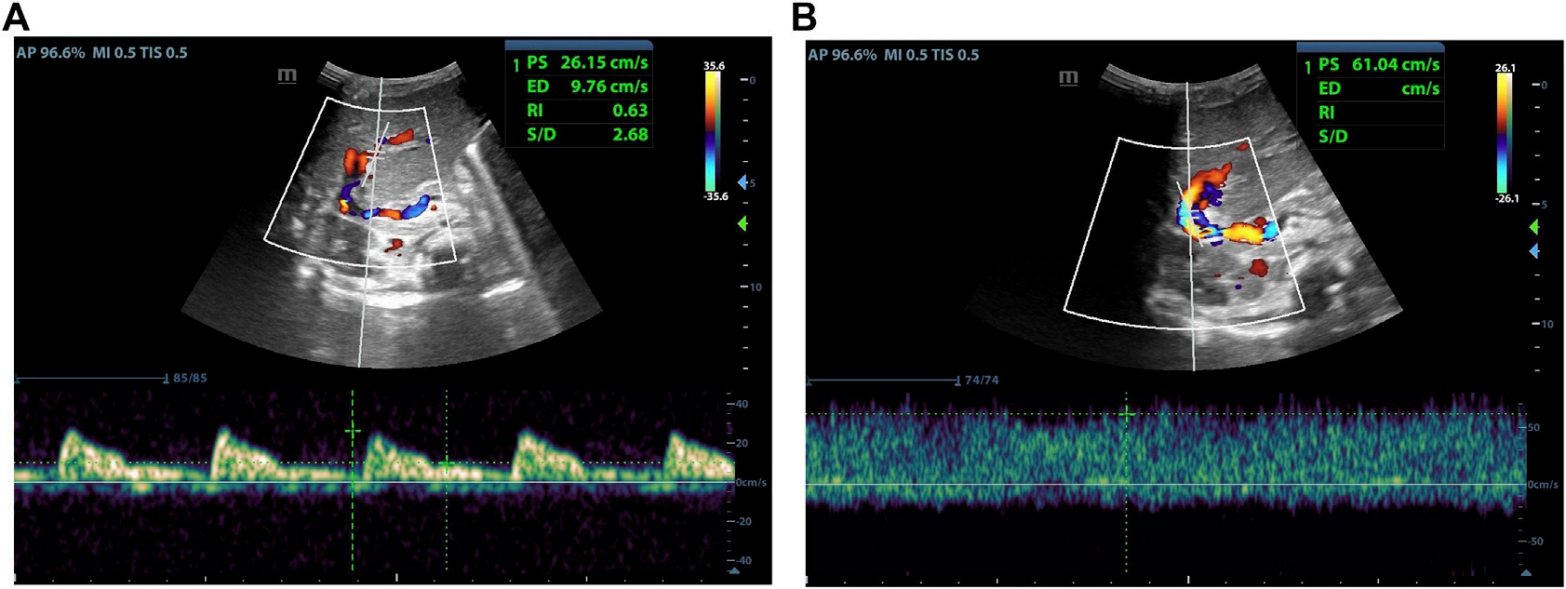
Male, 4 months old, 14 days after LDLT. **(A)** Hepatic artery flow spectrum. **(B)** Portal vein flow spectrum.

As indicated in Table 2, there was no statistical difference between POD7 and POD1 in values of PSV_HA_. At the same time, it was different between POD14 and POD1, and between POD30 and POD1. Compared with POD7, there was a statistical difference in values of PSV_HA_ at POD14 and POD30. There was no statistical difference between POD30 and POD14 in values of PSV_HA_. That is to say, PSV_HA_ decreased significantly from about 2 weeks after LDLT. As for PVV and RI_HA_, there was no statistical difference between each time point within 1 month after liver transplantation surgery, and the values changed gradually, showing an overall decrease trend (Figures 2–4).

**TABLE 2:** Hemodynamic parameters change at different time of the liver graft.

Time	HA	RI	PVV(cm/s)
	PSV(cm/s)		
POD1	53.10 ± 16.02	0.61 ± 0.05	30.80 ± 8.67
POD7	47.02 ± 10.05	0.59 ± 0.03	30.05 ± 8.80
POD14	42.29 ± 10.38^bc^	0.59 ± 0.05	28.39 ± 6.47
POD30	38.51 ± 7.63^bc^	0.59 ± 0.01	26.71 ± 7.93

Compared with POD1, ^b^ *p* < 0.01.

Compared with POD7, ^c^ *p* < 0.01.

POD1 postoperative day 1, POD7 postoperative day 7, POD14 postoperative day 14.

POD30 postoperative day 30.

## Discussion

Liver transplantation is currently the only effective treatment for end-stage liver diseases. LDLT has become the primary method of liver transplantation in infants and very young children, because it can provide suitable liver sources in time. Although the extensive development of Kasai surgery has saved some children’s lives with BA, if the Kasai operation is not timely or successful in rebuilding bile flow, about 70% of children still need liver transplantation due to repeated cholangitis or portal hypertension (Bezerra et al., 2018; Alexopoulos et al., 2017). Doppler ultrasonography plays a vital role in monitoring hemodynamic changes after liver transplantation. The physiological and pathological characteristics of infants and young children are more complicated than those of adults. Postoperative vascular complications are more likely to occur in infants. Knowing the hemodynamic changes in the grafts after LDLT helps identify early vascular complications, which is essential to improving the survival rate of recipients (Sanyal et al., 2014).

During the process of liver cirrhosis in children with BA, there are three changes (hepatocytes diffuse necrosis, fibrous tissue hyperplasia, and nodular regeneration of hepatocytes) that result in changes in the structure of the liver lobules, stenosis or occlusion of the liver sinusoids, as well as a higher resistance of the intrahepatic vascular bed. When the portal vein inflow is blocked, the pressure in the portal vein increases, and the blood flow velocity decreases. The obstruction of the portal flow promotes the opening of collateral vessels, and part of the portal flow will flow into the systemic venous system. As a result, the portal flow decreases, and the blood flow velocity is reduced.

Due to biliary liver cirrhosis, preoperative Doppler ultrasound in this cohort of BA children showed that the hepatic artery flow velocity and the RI were higher (Table 1). The more severe the degree of liver cirrhosis, the portal flow velocity is likely to be lower due to higher resistance in the vascular bed. Consequently, the hepatic artery flow velocity is likely to be higher due to HABR, and the higher the resistance index. After liver transplantation, the high resistance state of the portal venous system is rapidly alleviated, and the PVF and PVV increased. Meanwhile, the hepatic artery blood flow and flow velocity decrease due to HABR (Houssin et al., 1989; Payen et al., 1990). If portal blood flow is reduced, the hepatic artery dilates (Liu et al., 2016), and the hepatic artery constricts if the portal flow increases (Jakab et al., 1995; Lautt et al., 1990). RI_HA_ may increase when the hepatic artery constricts or spasms early after liver transplantation (Gu et al., 2015; Eipel et al., 2010).

Compared with the first day after surgery, this study found no significant difference in hepatic arterial flow velocity within 1 week after the operation, and the PSV_HA_ significantly reduced about 2 weeks after the operation. It is consistent with the conclusions of some previous studies. For example, Jamieson et al. (2014) found that normal pediatric Doppler parameters at POD1 trended toward higher velocities and resistive indexes, and it usually gradually decreased to normal at 7–15 days after surgery. The blood flow in the liver graft underwent drastic changes in the early stage after LT, especially on the first day after surgery. The first 24 h after transplantation are referred to as the “hyperdynamic phase” (Sanyal et al., 2014), in which the transplanted liver demonstrates disordered circulatory patterns and hemodynamic instability. Due to the history of cirrhosis before the operation, portal hypertension has not recovered to a normal level; the splanchnic circulation shows a rapid change in the portal and arterial perfusion (Bolognesi et al., 2002; Han et al., 2014). So the portal vein velocity was faster during the first 24 h after surgery (Table 1). Afterwards, PSV_HA_ gradually decreased (Table 2). Our study shows no statistical difference in RI_HA_ between each time point within 1 month after surgery.

According to the RI_HA_ values on POD1, some recipients were grouped in high (RI ≥ 0.8), normal (0.5 ≤ RI < 0.8), and low groups (RI < 0.5), among which the normal group accounted for 62.2%. Another study found RI > 0.8 in nearly half of grafts immediately after liver transplantation (Sanyal et al., 2014). It has been reported that the RI_HA_ rises in the early postoperative period, and it is more likely to occur in the elderly (Gaspari et al., 2020; García-Criado et al., 2003). The increase is transitory, and it may be related to the hepatic artery spasm or anastomotic edema due to the high portal blood flow. The RI detected in our study on the first day after surgery was slightly higher than that in the following month, but the difference was not statistically significant. There were some common features in this cohort: 1) The infants in our study were very young (median age 5 months) and the systemic and hepatic circulations were not fully developed; 2) The vascular bed resistance of the transplanted liver quickly reduced after the transplantation, so the RI was high before the operation and decreased immediately after the operation; 3) The enrolled infants were all in good condition after the operation without early postoperative vascular complications.

One subject in this cohort had a stable condition during the first week after the operation. On the POD7, the child underwent an ultrasound examination where the hepatic artery PSV reached 1.67 m/s, and the RI was 0.84. It was the highest hepatic arterial flow velocity and RI recorded 2 weeks after the operation. The clinical manifestations, the laboratory test of serum biochemical indicators for the liver function, and the patency of blood vessels were normal in subsequent ultrasound examinations. And the child was followed up for 18 months without abnormalities. Therefore, it is speculated that this transient high resistance index may be related to vasospasm during the ultrasound examination.

This study showed that the portal vein velocity declined slowly in the first month after transplantation, and there was no statistical difference at different time points after transplantation (1st day, 7th day, 14th day, and 30th day). Some long-term follow-up studies found that it took about 1–4 years for the portal vein velocity to return to normal (Han et al., 2014). It can be concluded that the recovery of portal vein hemodynamics is a gradual and long-term process. Some studies found the high portal blood flow velocities seen immediately postoperative are often transient and decrease on follow-up scans.

The study concentrated only on the early postoperative period, information of more long-term prognosis was lacking. In addition, since the age of onset of congenital biliary atresia is mainly around 1 year old, it added to the difficulty in the examination for infants and young children. In addition, the diameter of the hepatic artery in infants is very small therefore it was a challenge to obtain ideal hepatic blood flow parameters when they are not in a calm state, resulting in a lack of comparison with normal reference values.

Nevertheless, the study can form a solid base for computational models for the variations of hemodynamics after liver transplantation in infants, where data are still scant. General speaking, a transplanted liver graft into an infant should already satisfy the requirement for the minimum weight or parenchyma volume. However, abnormal portal perfusion and hepatic venous drainage are a threat to graft survival. Simulations for the pre- and post-transplantation hepatic flow, surgical repair and graft growth are valuable for surgical training and research.

## Conclusion

In this retrospective study of liver hemodynamics before and 1 month after liver transplantation, we found the PSV_HA_ and RI_HA_ on the first day after liver transplantation was significantly lower, and the PVV was higher than those before surgery. The PSV_HA_ began to decrease significantly at about 2 weeks after the operation, and the PVV and RI did not change significantly after 1 month. The results could be used for future LT studies as well as biomechanics models for the blood flow in infant livers.

## Data Availability

The original contributions presented in the study are included in the article/Supplementary Material, further inquiries can be directed to the corresponding authors.

## References

[B1] Abdelaziz O. Attia H. (2016). Doppler ultrasonography in living donor liver transplantation recipients: Intra- and post-operative vascular complications. World J. Gastroenterol. 22, 6145–6172. 10.3748/wjg.v22.i27.6145 27468207 PMC4945976

[B2] Alexopoulos S. P. Nekrasov V. Cao S. Groshen S. Kaur N. Genyk Y. S. (2017). Effects of recipient size and allograft type on pediatric liver transplantation for biliary atresia. Liver Transpl. 23, 221–233. 10.1002/lt.24675 27862929

[B3] Bezerra J. A. Wells R. G. Mack C. L. Karpen S. J. Hoofnagle J. H. Doo E. (2018). Biliary atresia: Clinical and research challenges for the twenty-first century. Hepatology 68, 1163–1173. 10.1002/hep.29905 29604222 PMC6167205

[B4] Bolognesi M. Sacerdoti D. Bombonato G. Merkel C. Sartori G. Merenda R. (2002). Change in portal flow after liver transplantation: Effect on hepatic arterial resistance indices and role of spleen size. Hepatology 35, 601–608. 10.1053/jhep.2002.31352 11870373

[B5] Chen C. L. Concejero A. Wang C. C. Wang S. H. Lin C. C. Liu Y. W. (2006). Living donor liver transplantation for biliary atresia: A single-center experience with first 100 cases. Am. J. Transpl. 6, 2672–2679. 10.1111/j.1600-6143.2006.01528.x 16939513

[B6] Eipel C. Abshagen K. Vollmar B. (2010). Regulation of hepatic blood flow: The hepatic arterial buffer response revisited. World J. Gastroenterol. 16, 6046. 10.3748/wjg.v16.i48.6046 21182219 PMC3012579

[B7] García-Criado A. Gilabert R. Salmerón J. M. Nicolau C. Vilana R. Bianchi L. (2003). Significance of and contributing factors for a high resistive index on Doppler sonography of the hepatic artery immediately after surgery: Prognostic implications for liver transplant recipients. Am. J. Roentgenol. 181, 831–838. 10.2214/ajr.181.3.1810831 12933490

[B8] Gaspari R. Teofili L. Mignani V. Franco A. Valentini C. G. Cutuli S. L. (2020). Duplex Doppler evidence of high hepatic artery resistive index after liver transplantation: Role of portal hypertension and clinical impact. Dig. Liver Dis. 52, 301–307. 10.1016/j.dld.2019.10.017 31806469

[B9] Gu L. Fang H. Li F. Zhang S. Shen C. Han L. (2015). Impact of hepatic arterial hemodynamics in predicting early hepatic arterial thrombosis in pediatric recipients younger than three yr after living donor liver transplantation. Pediatr. Transpl. 19, 273–278. 10.1111/petr.12444 25693722

[B10] Han H. Liu R. Wang W. P. Ding H. Wen J. X. Lin X. Y. (2014). Postoperative haemodynamic changes in transplanted liver: Long-term follow-up with ultrasonography. J. Int. Med. Res. 42, 849–856. 10.1177/0300060514521153 24651994

[B11] Hartley J. L. Davenport M. Kelly D. A. (2009). Biliary atresia. Lancet 374, 1704–1713. 10.1016/s0140-6736(09)60946-6 19914515

[B12] Houssin D. Fratacci M. Dupuy P. Vigouroux C. Gatecel C. Payen D. (1989). One week of monitoring of portal and hepatic arterial blood flow after liver transplantation using implantable pulsed Doppler microprobes. Transpl. Proc. 21, 2277–2278. 2652737

[B13] Jakab F. Ráth Z. Schmal F. Nagy P. Faller J. (1995). The interaction between hepatic arterial and portal venous blood flows; simultaneous measurement by transit time ultrasonic volume flowmetry. Hepatogastroenterology. 42, 18–21. 7782028

[B14] Jamieson L. H. Arys B. Low G. Bhargava R. Kumbla S. Jaremko J. L. (2014). Doppler ultrasound velocities and resistive indexes immediately after pediatric liver transplantation: Normal ranges and predictors of failure. Am. J. Roentgenol. 203, W110–W116. 10.2214/ajr.13.11685 24951222

[B15] Kelly D. A. Bucuvalas J. C. Alonso E. M. Karpen S. J. Allen U. Green M. (2013). Long-term medical management of the pediatric patient after liver transplantation: 2013 practice guideline by the American association for the study of liver diseases and the American society of transplantation. Liver Transpl. 19, 798–825. 10.1002/lt.23697 23836431

[B16] Lautt W. W. Legare D. J. Ezzat W. R. (1990). Quantitation of the hepatic arterial buffer response to graded changes in portal blood flow. Gastroenterology 98, 1024–1028. 10.1016/0016-5085(90)90029-z 2311859

[B17] Liu C. Song J. L. Lu W. S. Yang J. Y. Jiang L. Yan L. N. (2016). Hepatic arterial buffer response maintains the homeostasis of graft hemodynamics in patient receiving living donor liver transplantation. Dig. Dis. Sci. 61, 464–473. 10.1007/s10620-015-3881-8 26441282

[B18] Low G. Crockett A. M. Leung K. Walji A. H. Patel V. H. Shapiro A. M. (2013). Imaging of vascular complications and their consequences following transplantation in the abdomen. Radiographics 33, 633–652. 10.1148/rg.333125728 23674767

[B19] Payen D. M. Fratacci M. D. Dupuy P. Gatecel C. Vigouroux C. Ozier Y. (1990). Portal and hepatic arterial blood flow measurements of human transplanted liver by implanted Doppler probes: Interest for early complications and nutrition. Surgery 107, 417–427. 2181716

[B20] Piardi T. Lhuaire M. Bruno O. Memeo R. Pessaux P. Kianmanesh R. (2016). Vascular complications following liver transplantation: A literature review of advances in 2015. World J. Hepatol. 8, 36–57. 10.4254/wjh.v8.i1.36 26783420 PMC4705452

[B21] Sanada Y. Mizuta K. Urahashi T. Ihara Y. Wakiya T. Okada N. (2011). Hepatic arterial buffer response after pediatric living donor liver transplantation: Report of a case. Transpl. Proc. 43, 4019–4024. 10.1016/j.transproceed.2011.08.094 22172893

[B22] Sanyal R. Zarzour J. G. Ganeshan D. M. Bhargava P. Lall C. G. Little M. D. (2014). Postoperative doppler evaluation of liver transplants. Indian J. Radiol. Imaging 24, 360–366. 10.4103/0971-3026.143898 25489129 PMC4247505

[B23] Someda H. Moriyasu F. Fujimoto M. Hamato N. Nabeshima M. Nishikawa K. (1995). Vascular complications in living related liver transplantation detected with intraoperative and postoperative Doppler US. J. Hepatology 22, 623–632. 10.1016/0168-8278(95)80218-5 7560856

[B24] Tang Y. Zhang G. Kong W. Yu H. Niu N. Liu J. (2021). Pediatric living donor left lateral segment liver transplantation for biliary atresia: Doppler ultrasound findings in early postoperative period. Jpn. J. Radiol. 39, 367–375. 10.1007/s11604-020-01067-4 33161495

[B25] Wan P. Xu D. Zhang J. Li Q. Zhang M. Chen X. (2016). Liver transplantation for biliary atresia: A nationwide investigation from 1996 to 2013 in mainland China. Pediatr. Transpl. 20, 1051–1059. 10.1111/petr.12750 27368158

[B26] Wang Q. Yan L. N. Zhang M. M. Wang W. T. Zhao J. C. Pu C. L. (2013). The pre-Kasai procedure in living donor liver transplantation for children with biliary atresia. Hepatobiliary Pancreat. Dis. Int. 12, 47–53. 10.1016/s1499-3872(13)60005-3 23392798

