# Corrigendum: Doppler evaluation of hepatic hemodynamics after living donor liver transplantation in infants

**DOI:** 10.3389/fbioe.2022.1036918

**Published:** 2022-09-30

**Authors:** Xiping Chen, Huan Xiao, Chunjiang Yang, Jingyu Chen, Yang Gao, Yi Tang, Xiaojuan Ji

**Affiliations:** ^1^ Department of Ultrasound, National Clinical Research Center for Child Health and Disorders, Ministry of Education Key Laboratory of Child Development and Disorders, Children’s Hospital of Chongqing Medical University, Chongqing, China; ^2^ Department of Ultrasound, Chongqing General Hospital, Chongqing, China

**Keywords:** Doppler ultrasound, hemodynamics, liver transplantation, hepatic artery, portal vein, child

In the published article, there was an error in **Supplementary Figure 2A**, an incorrect image was used. The updated **Supplementary Figure 2A** appears in the **Supplementary Material**.

The authors apologize for this error and state that this does not change the scientific conclusion of the article in any way. The original article has been updated.

