# Supplementary figures and images for: Doppler evaluation of hepatic hemodynamics after living donor liver transplantation in infants
Source: Front Bioeng Biotechnol. 2022 Aug 11;10:903385. doi: 10.3389/fbioe.2022.903385 (PMC9402890; doi:10.3389/fbioe.2022.903385)

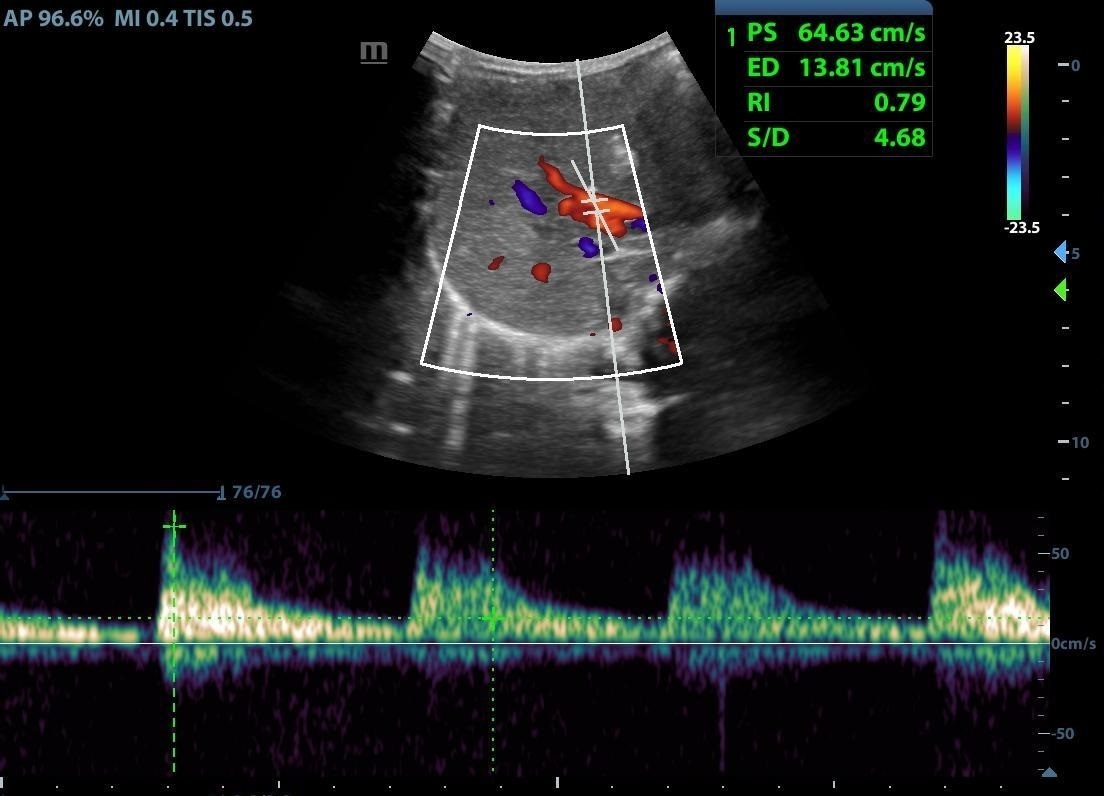

Supplement: Supplementary file 1 [file Presentation1.ZIP › Figures/1A.jpg]

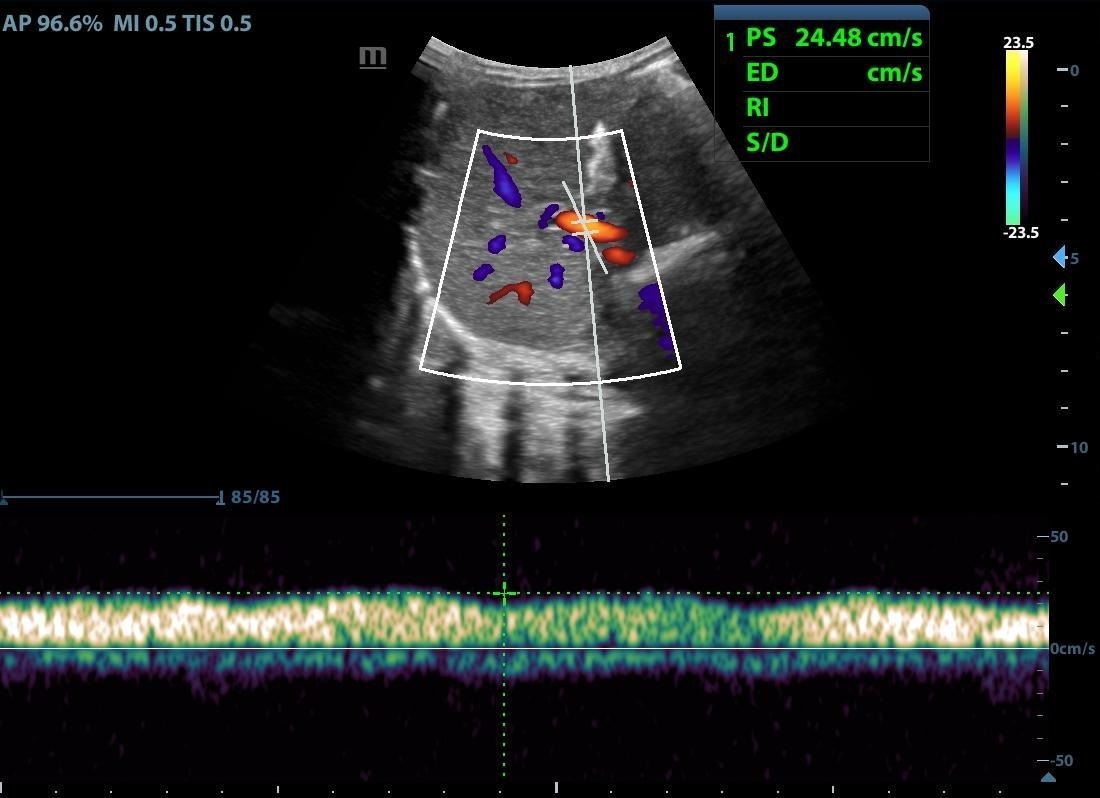

Supplement: Supplementary file 1 [file Presentation1.ZIP › Figures/1B.jpg]

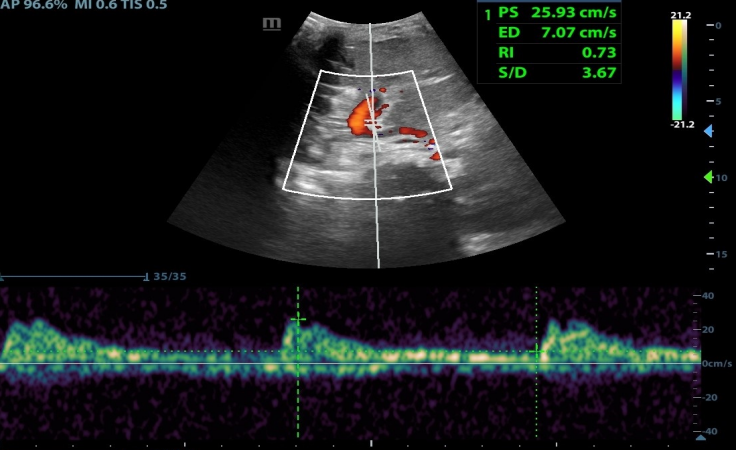

Supplement: Supplementary file 1 [file Presentation1.ZIP › Figures/2A.png]

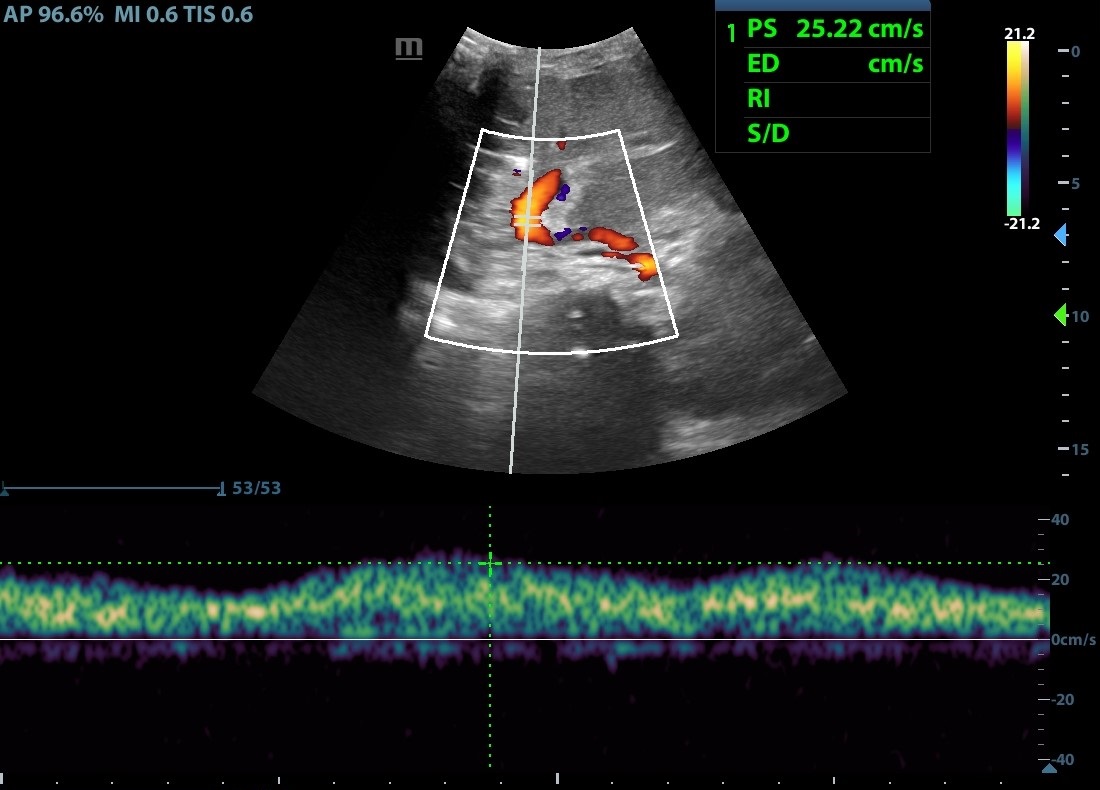

Supplement: Supplementary file 1 [file Presentation1.ZIP › Figures/2B.jpg]

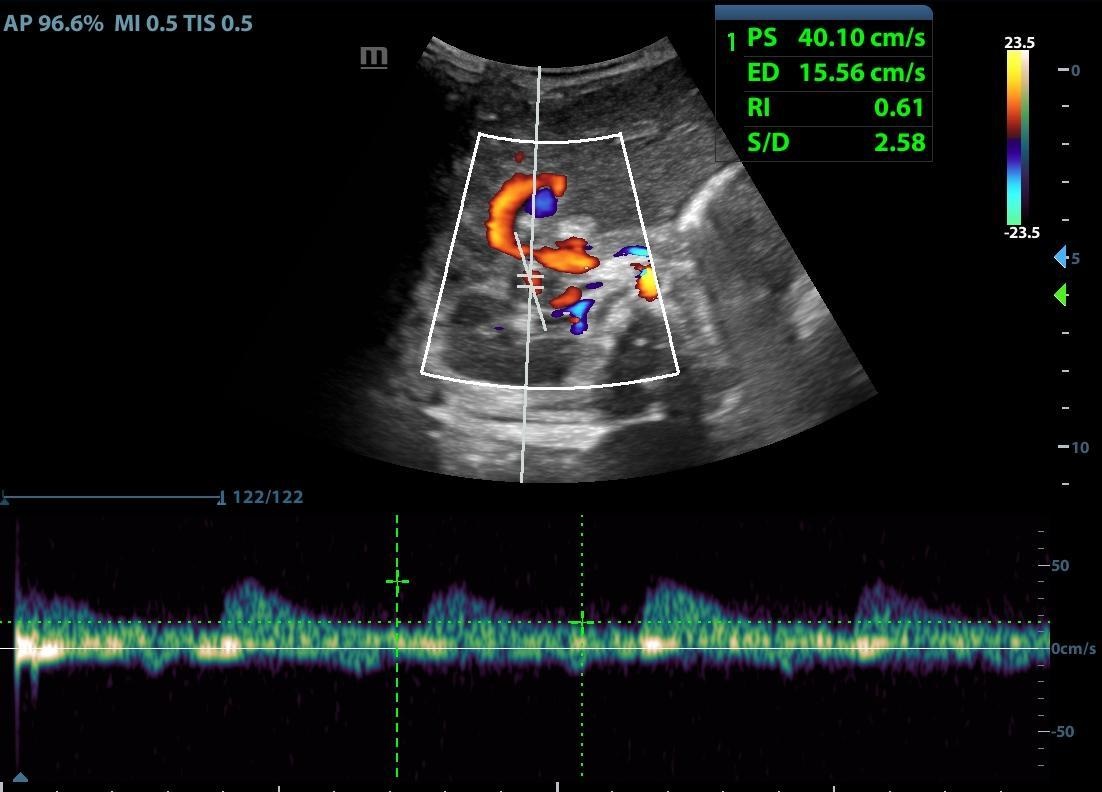

Supplement: Supplementary file 1 [file Presentation1.ZIP › Figures/3A.jpg]

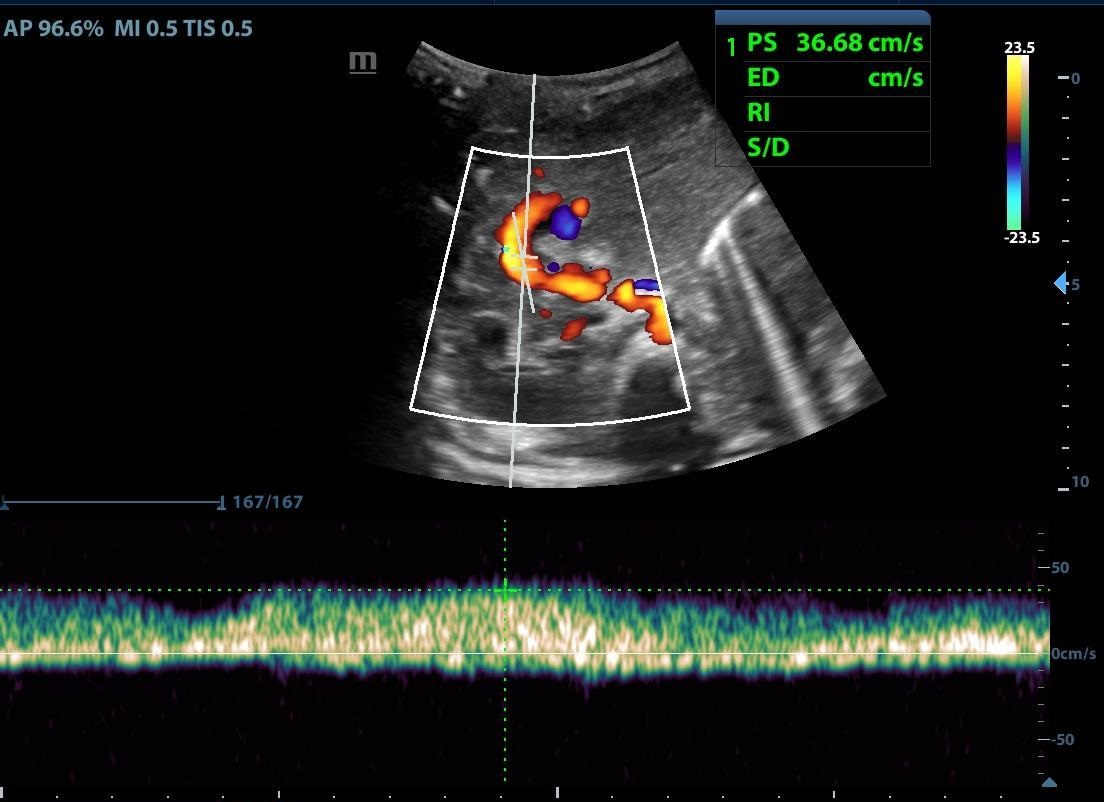

Supplement: Supplementary file 1 [file Presentation1.ZIP › Figures/3B.jpg]

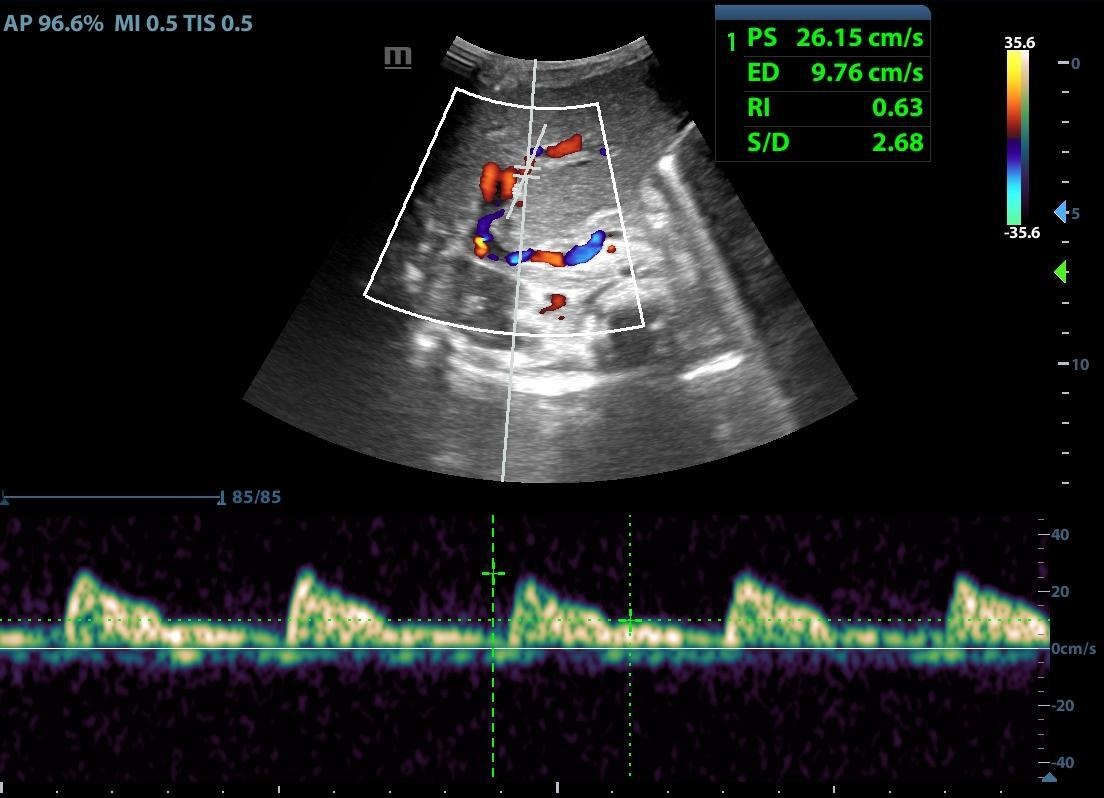

Supplement: Supplementary file 1 [file Presentation1.ZIP › Figures/4A.jpg]

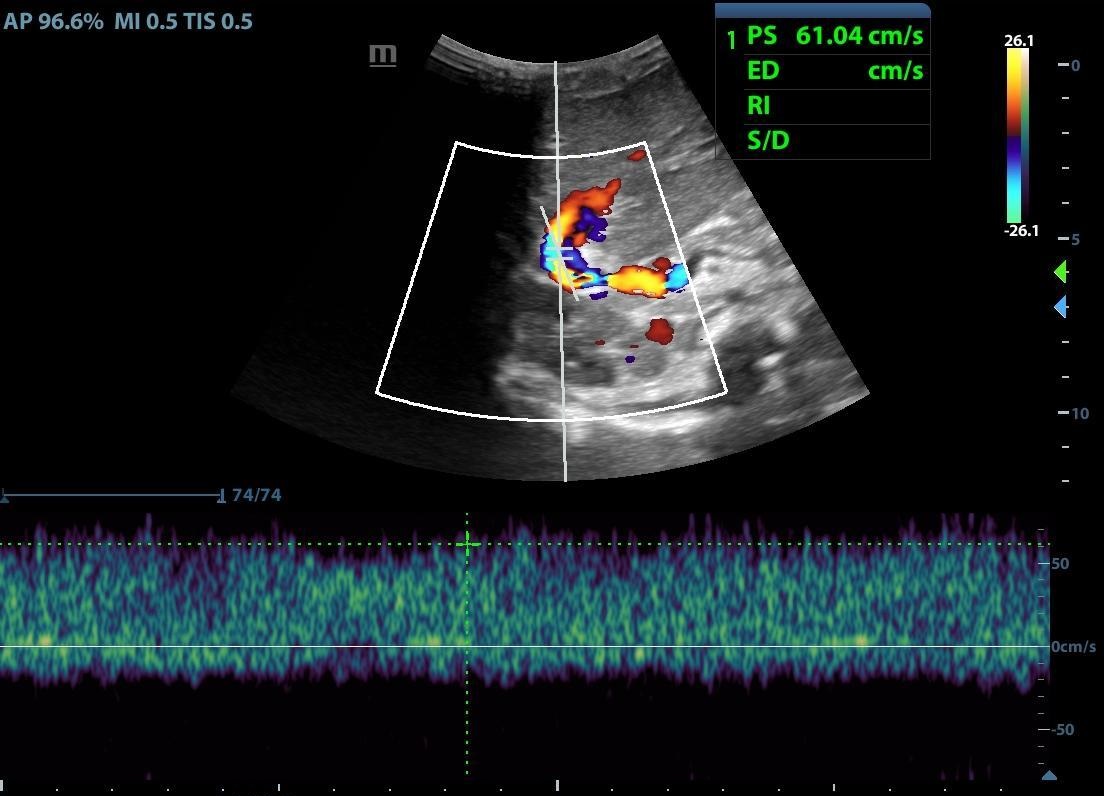

Supplement: Supplementary file 1 [file Presentation1.ZIP › Figures/4B.jpg]
